# A prognostic model based on seven immune-related genes predicts the overall survival of patients with hepatocellular carcinoma

**DOI:** 10.1186/s13040-021-00261-y

**Published:** 2021-05-07

**Authors:** Qian Yan, Wenjiang Zheng, Boqing Wang, Baoqian Ye, Huiyan Luo, Xinqian Yang, Ping Zhang, Xiongwen Wang

**Affiliations:** 1 The First Clinical Medical School, Guangzhou University of Chinese Medicine, Guangzhou, China; 2 Department of Oncology, The First Affiliated Hospital, Guangzhou University of Chinese Medicine, Guangzhou, China

**Keywords:** Hepatocellular carcinoma, Immune-related genes, Prognostic model, Nomogram, Immune infiltration

## Abstract

**Background:**

Hepatocellular carcinoma (HCC) is a disease with a high incidence and a poor prognosis. Growing amounts of evidence have shown that the immune system plays a critical role in the biological processes of HCC such as progression, recurrence, and metastasis, and some have discussed using it as a weapon against a variety of cancers. However, the impact of immune-related genes (IRGs) on the prognosis of HCC remains unclear.

**Methods:**

Based on The Cancer Gene Atlas (TCGA) and Immunology Database and Analysis Portal (ImmPort) datasets, we integrated the ribonucleic acid (RNA) sequencing profiles of 424 HCC patients with IRGs to calculate immune-related differentially expressed genes (DEGs). Survival analysis was used to establish a prognostic model of survival- and immune-related DEGs. Based on genomic and clinicopathological data, we constructed a nomogram to predict the prognosis of HCC patients. Gene set enrichment analysis further clarified the signalling pathways of the high-risk and low-risk groups constructed based on the IRGs in HCC. Next, we evaluated the correlation between the risk score and the infiltration of immune cells, and finally, we validated the prognostic performance of this model in the GSE14520 dataset.

**Results:**

A total of 100 immune-related DEGs were significantly associated with the clinical outcomes of patients with HCC. We performed univariate and multivariate least absolute shrinkage and selection operator (Lasso) regression analyses on these genes to construct a prognostic model of seven IRGs (Fatty Acid Binding Protein 6 (*FABP6*), Microtubule-Associated Protein Tau (*MAPT*), Baculoviral IAP Repeat Containing 5 (*BIRC5*), Plexin-A1 (*PLXNA1*), Secreted Phosphoprotein 1 (*SPP1*), Stanniocalcin 2 (*STC2*) and Chondroitin Sulfate Proteoglycan 5 (*CSPG5*)), which showed better prognostic performance than the tumour/node/metastasis (TNM) staging system. Moreover, we constructed a regulatory network related to transcription factors (TFs) that further unravelled the regulatory mechanisms of these genes. According to the median value of the risk score, the entire TCGA cohort was divided into high-risk and low-risk groups, and the low-risk group had a better overall survival (OS) rate. To predict the OS rate of HCC, we established a gene- and clinical factor-related nomogram. The receiver operating characteristic (ROC) curve, concordance index (C-index) and calibration curve showed that this model had moderate accuracy. The correlation analysis between the risk score and the infiltration of six common types of immune cells showed that the model could reflect the state of the immune microenvironment in HCC tumours.

**Conclusion:**

Our IRG prognostic model was shown to have value in the monitoring, treatment, and prognostic assessment of HCC patients and could be used as a survival prediction tool in the near future.

## Introduction

Ranking sixth in worldwide incidence, primary liver cancer (PLC) is the fourth-leading cause of cancer-related mortality [1]. Hepatocellular carcinoma (HCC), the most common pathological type of PLC, accounts for approximately 90% of reported cases [2–5]. Hepatitis B and C viruses are the biggest risk factors for HCC [6]. Application of the hepatitis B virus vaccine has caused the incidence of HCC to decline [7]. Leaving aside patients who are diagnosed at an early stage or eligible for potentially curative therapies, treatment for advanced HCC is limited due to its heterogeneity, and the overall prognosis of HCC patients is still unsatisfactory [8, 9].

Cancer immunotherapy has contributed to personalized medicine, with substantial clinical benefit against advanced disease [10–15]. Current immune checkpoint inhibitors show surprising potential effectiveness against HCC [16, 17]. Indeed, the liver is a central immunological organ with a high density of myeloid and lymphoid immune cells [17, 18]. Immune cells are widespread in the tumor microenvironment (TME) [19, 20], wherein interaction between tumor cells and immune cells is extremely important to maintaining the dynamic balance of normal tissues and tumor growth; this process is closely related to the occurrence, progression, and prognosis of cancer [21]. Meanwhile, inflammatory reaction plays a decisive role at different stages of tumor development. It also affects immune monitoring and response to treatment and promotes the occurrence and development of tumours to varying degrees [22]. Since HCC often arises in the setting of chronic liver inflammation [5, 23] and might be responsive to novel immunotherapies, people infected with hepatitis B or C viruses are at high risk of HCC [24]. While several studies have supported the importance of immunology in HCC, the exact molecular mechanisms still remain unknown, particularly for combinations of immune cells forming a TME [25] and for immunogenomic effects [26]. With the advent of multi-dimensional, large-scale high-throughput analyses, cancer researchers have been able to identify culpable biomarkers for tumour prognosis and prediction [27–30]. Long et al. explored the prognostic value of immune-related genes (IRGs) linked to *TP53* status in order to improve the prognoses of HCC patients [31]. Moeinia et al. analysed the expression profiles of 392 early-stage non-tumour liver tissues from HCC patients and liver tissues from HCC-free cirrhosis patients, identified possible regulatory changes in the expression of IRGs in HCC, and further verified the accuracy of this conclusion through experiments. This gene expression pattern is related to the risk of PLC in cirrhosis patients [32]. Liang S et al. proposed that after liver injury, the molecular pattern related to the release of hepatocytes would activate liver tumour-associated macrophages (TAMs), thus producing cytokines to promote tumour development [33]. However, the clinical relevance and prognostic significance of IRGs in HCC have yet to be comprehensively explored.

Our study aimed to better appreciate the potential clinical utility of IRGs prognostic stratification and develop a new IRG-based immune prognostic model (IPM). We systematically investigated the expression status from The Cancer Gene Atlas (TCGA, https://cancergenome.nih.gov/) database and prognostic landscape of IRGs, constructed a genomic–clinicopathological model for these patients and validate it in Gene Expression Omnibus (GEO, https://www.ncbi.nlm.nih.gov/geo/). Moreover, underlying regulatory mechanisms have been explored by bioinformatics analysis. The results of this study could help provide a more complete understanding and more-precise immunotherapy for HCC.

## Materials and methods

### HCC datasets and preprocessing

As TCGA and GEO databases both are landmark cancer genome projects that are publicly available to any researcher, our research did not require the approval of an ethics committee. After downloading data from transcriptome messenger ribonucleic-acid (mRNA) expression profiles and the clinical information of HCC patients from the TCGA and GEO website, we ultimately obtained a dataset of 374 HCC and 50 para-tumor samples [34] as a training dataset, 225 HCC tissues and 220 adjacent non-tumour samples (GPL3921) in GSE14520 dataset as a test dataset [35].

Also, we obtained a list of IRGs from the Immunology Database and Analysis Portal (ImmPort, https://www.immport.org/shared/home). This is one of the largest open source repositories of human immunological data at the subject level, providing data on clinical and mechanistic studies of human subjects and immunological studies of model organisms [36]. The integrated analysis of these databases, which reveals new insights into the comprehensive analysis yielded by the combination of mass spectrometry staining and tumour molecular profiling, could become a useful resource on the regulation of tumour-related genes.

### Identification, normalization, and elucidation of differentially expressed genes (DEGs) and immune-related genes (IRGs)

We used the limma package in R software (version 3.5.3; R Foundation for Statistical Computing) to calculate genes in common between HCC and para-tumour tissue [37]. The absolute value of log fold change (FC) was ≥2, and adjusted *P* < 0.05 was the cutoff value. We screened DEGs between the two groups and depicted the results in a heatmap and volcano plot. Then, we use the combat function in the sva package in R software to remove batch effects and batch corrections on the gene expression data between the training and test group [38]. By combining DEGs and IRGs, we obtained the intersection of IRGs involved in HCC pathogenesis, and all of the IRGs were listed in GSE14520 dataset, too. To explore the potential functions and possible pathways of these IRGs, we further analysed the differentially expressed IRGs via gene ontology (GO) and Kyoto Encyclopedia of Genes and Genomes (KEGG) pathway analysis, enabled by the clusterProfiler package in R software [39].

### Screening of prognosis-specific IRGs

We combined and analysed the patients’ clinical information and the gene expression of IRGs, using OS as the outcome index. Samples with an OS time of less than 30 days and incomplete clinical information were omitted, and we finally retained 343 samples in the TCGA dataset and 221 samples in the GSE14520 dataset to construct the model. Detailed epidemiological information of the two cohorts is displayed in Table 1. The significance level of univariate Cox regression analysis was set to *P* < 0.05 and displayed in the form of a forest plot.

**Table 1:** Clinical information in training and validation groups

Characteristics	Training group (*N* = 343)		Testing group (*N* = 221)	
	High risk	Low risk	High risk	Low risk
Age				
<60	78	79	119	59
≥ 60	93	93	23	20
Gender				
Male	117	116	125	66
Female	54	56	17	13
ALT (>/<=50 U/L)				
high	–	–	60	31
low	–	–	82	47
Unknown			0	1
Main Tumor Size (>/<=5 cm)				
Large	–	–	57	23
Small	–	–	84	56
Unknown	–	–	1	0
Multinodular				
Y	–	–	34	11
N	–	–	108	68
Cirrhosis				
Y	–	–	133	70
N	–	–	9	9
Unknown	–	–		
CLIP staging				
0	–	–	54	53
1	–	–	47	27
2	–	–	30	5
3	–	–	7	2
4	–	–	1	2
5	–	–	1	0
9	–	–	2	0
Grade				
G1	15	38	–	–
G2	74	87	–	–
G3	71	41	–	–
G4	9	3	–	–
Unknown	2	3	–	–
TNM Stage				
T1	58	104	45	48
T2	49	28	54	23
T3	50	30	41	8
T4	2	1	2	0
Unknown	12	9		
T				
T1	61	107	–	–
T2	55	29	–	–
T3	46	29	–	–
T4	9	4	–	–
Unknown	0	3	–	–
N				
N0	121	118	–	–
N1	3	0	–	–
Unknown	47	54	–	–
M				
M0	125	120	–	–
M1	2	1	–	–
Unknown	44	51	–	–
BCLC staging				
0	–	–	14	6
1	–	–	84	64
2	–	–	18	4
3	–	–	24	5
9	–	–	2	0
AFP (>/<=300 ng/ml)				
High	–	–	70	30
Low	–	–	70	48
Unknown	–	–	2	1

Abbreviations: *TCGA-LIHC* The Cancer Genome Atlas, Liver Hepatocellular Carcinoma; *ALT* Alanine Transaminase; *CLIP staging* Cancer of the Liver Italian Program staging; *TNM Stage*: Tumor Node Metastasis stage; *BCLC staging* Barcelona Clinic Liver Cancer staging; *AFP* Alpha Fetoprotein

### Transcription factor (TF) regulatory network

TF protein are critical regulators of gene switches [40]. The Cistrome Cancer database (http://cistrome.org/CistromeCancer/CancerTarget/) combines the cancer genomics data in TCGA with the chromatin analysis data in the Cistrome Data Browser, enabling cancer researchers to explore how TFs regulate the degree of gene expression [41]. To explore the regulatory mechanisms of prognosis-related IRGs, we built a regulatory network covering differentially expressed TFs and IRGs using Cytoscape software version 3.7.1 (Cytoscape Consortium; https://cytoscape.org/) [42]. We also conducted protein–protein interaction (PPI) analysis using the Search Tool for the Retrieval of Interacting Genes/Proteins (STRING; STRING Consortium; https://string-db.org/) to evaluate interactions among all of the TFs. Using the cytoHubba package in Cytoscape, we also performed topological analysis of these key TFs and ranked the top 10 by the “degree” criterion [43].

### Construction of IPMs and validation model

The glmnet package was utilized to build a multivariate least absolute shrinkage and selection operator (Lasso) Cox proportional hazards regression model, and the cv.glmnet function was used to create 1000 random iterations. We obtained the best modelling parameters through 10-fold cross-validation and the default “deviance”, hence constructing an IPM of the IRGs [44]. The calculation formula was as follows:

$$ risk\kern0.5em score=\sum \limits_{n=1}^{\infty}\left({\beta}_n\times {\in}_n\right) $$

where β represents the weight of each gene, and ∈ is the standardized expression value of each gene. According to the median value of the risk score, the entire TCGA dataset was divided into two groups. We also divided the GSE14520 data set into high- and low- risk groups according to the median in the training set. We applied Kaplan-Meier (K-M) survival analyses curves to see if there were any differences between these two groups. At the same time, we displayed the risk scores, survival status, and gene expression levels of patients in the high-risk and low-risk groups.

### Construction and validation of the prognosis-related nomogram

We built 1-, 3-, and 5-year nomograms of key genes in the IPM using the rms packages in R software. To evaluate the sensitivity and specificity of our IPM, we drew time-dependent receiver operating characteristic (ROCs) curves and calibration curves, and calculated a concordance index (C-index) using the survivalROC installation package in R software [45]. When the C-index is between 0.5–0.7, it proves that the prognostic performance of the model is statistically acceptable; and when C-index > 0.7, we considered the predictive power of our model has a high degree of discrimination [46].

### Correlations between risk score and clinical features

Similarly, we analysed the significance of risk score correlated with clinical factors in multivariate and univariate analyses, and constructed a nomogram to evaluate practical-application value of the nomogram. The clinical factors in the training set include age, gender, TNM staging and grade; the clinical information in the testing set include gender, age, alanine transaminase (ALT) (>/<=50 U/L), main tumour size (>/<=5 cm), multinodular, cirrhosis, tumour node metastasis (TNM) staging, Barcelona Clinic Liver Cancer (BCLC) staging, Cancer of the Liver Italian Program (CLIP) staging and alpha fetoprotein (AFP) (>/<=300 ng/ml). In addition, the time-independent ROC curve and C-index value were used to assess its prognostic performance, too. We further analysed the correlation of various clinical factors with gene expression levels and risk scores in the IPM.

### Gene set enrichment analysis

GSEA v4.0.1 software was used to further identify different biological processes between the low-risk and high-risk groups constructed by the seven IRGs in HCC. We carried out gene set enrichment analysis (GSEA, https://www.gsea-msigdb.org/gsea/index.jsp) to explore the enriched items of the two groups [47] and “c2.all.v7.4.symbols.gmt” was chosen as the reference gene set. *P* < 0.05 and false discovery rate < 0.25 were used as the screening criteria.

### Relationship between risk sore and immune cell infiltration

The Tumour Immune Estimation Resource online database (TIMER, http://cistrome.org/TIMER/) can estimate the infiltration abundance of six common types of immune cells-B cells, Cluster of Differentiation 4-positive (CD4^+^) T cells, Cluster of Differentiation 8-positive (CD8^+^) T cells, neutrophils, TAMs, and dendritic cells (DCs)-and provide a comprehensive resource on immune infiltration of various cancer types [17]. Hence, we performed Pearson correlation analysis between risk score and the content of six types of immune cells.

### Verification of immune-related signatures

We analysed genetic alterations in seven IRGs associated with prognosis. The data were obtained from the cBio Cancer Genomics Portal (cBioPortal, http://www.cbioportal.org/), which is of great utility in exploring multidimensional genomic information [48]. The human protein atlas project (HPA, https://www.proteinatlas.org/) is used to evaluate the protein level differences of each IRGs [49]. To obtain the effect on HCC survival of high and low expression of these genes in HCC, we input them into the Kaplan Meier Plotter (K-M, https://kmplot.com/analysis/), a website providing gene chips and RNA sequencing data sources from the GEO and TCGA for several cancers [48, 50]. *P* < 0.05 was considered to be statistically significant. We calculated OS, disease-free survival (DFS), progression-free survival (PFS), and relapse-free survival (RFS) rates for HCC.

### Statistical analysis

Most of the statistical analyses was performed using R software and online databases. PPI network analysis was completed and the diagram of mechanism regulation between TFs and IRGs was created using Cytoscape. Pearson correlation analysis was used to analyse the correlation between risk score and clinical factors and the degree of immune cell infiltration. In addition, we used the cBioPortal and K-M Plotter to analyse the genetic changes and survival differences of genes, respectively.

## Results

### Differentially expressed OS-related DEGs in HCC

**Fig. 1:**
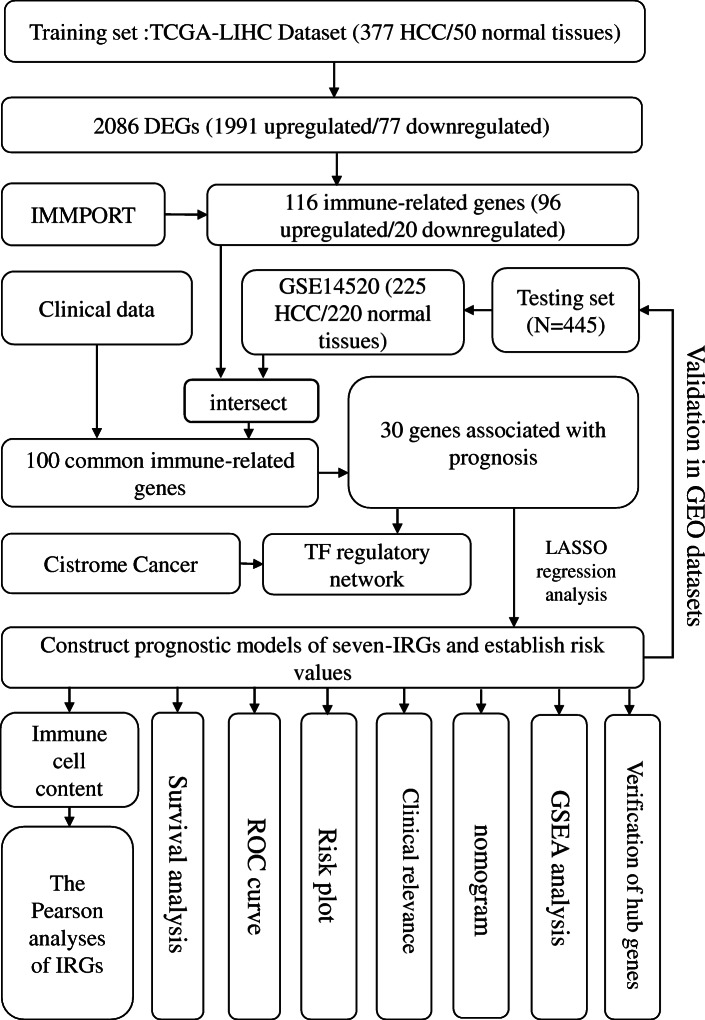
Flowchart presenting the process of establishing the seven-gene signature and prognostic nomogram for HCC. Abbreviations: HCC: hepatocellular carcinoma; TCGA-LIHC: The Cancer Genome Atlas, Liver Hepatocellular Carcinoma; GEO: Gene Expression Omnibus; IMMPORT: Immunology Database and Analysis Portal; DEG: differentially expressed gene; TF: transcription factor; ROC: Receiver operating characteristic; IRG: immune-related gene; LASSO: Least Absolute Shrinkage and Selection Operator; GSEA: Gene Set Enrichment Analysis

**Fig. 2:**
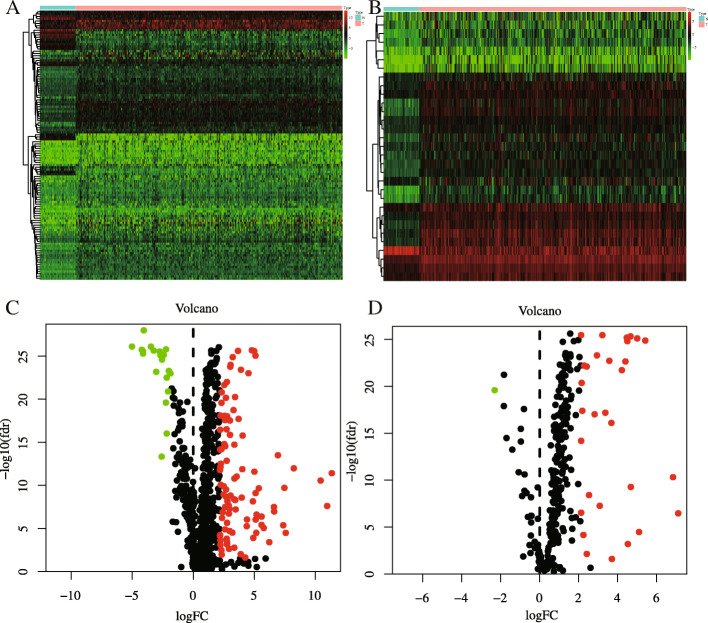
The filter results of differentially expressed immune related genes (IRGs) and transcription factors (TFs) between 374 hepatocellular carcinoma (HCC) and 50 para-tumor samples. **a** Heatmap and Volcano plot (**c**) of differentially expressed IRGs; (**b**) Heatmap and volcano plot (D) of differentially expressed TFs. Green and red dots separately represent low and high expression of IRGs and TFs in HCC, and black dots represent genes that are not differentially expressed

The flowchart in Fig. 1 clearly illustrates our analytic process. According to our screening criteria (|log FC| > 2, adjusted *P* < 0.05), the limma package identified 2068 DEGs in common between HCC and normal liver tissue. These DEGs included 1991 upregulated and 77 downregulated genes (Fig. 2a, d). From this group of genes, we extracted 116 differentially expressed IRGs, including 96 upregulated and 20 downregulated genes (Fig. 2b, e). Finally, we obtained 100 IRGs that exist both in the TCGA and GSE14520 dataset for model construction.

**Fig. 3:**
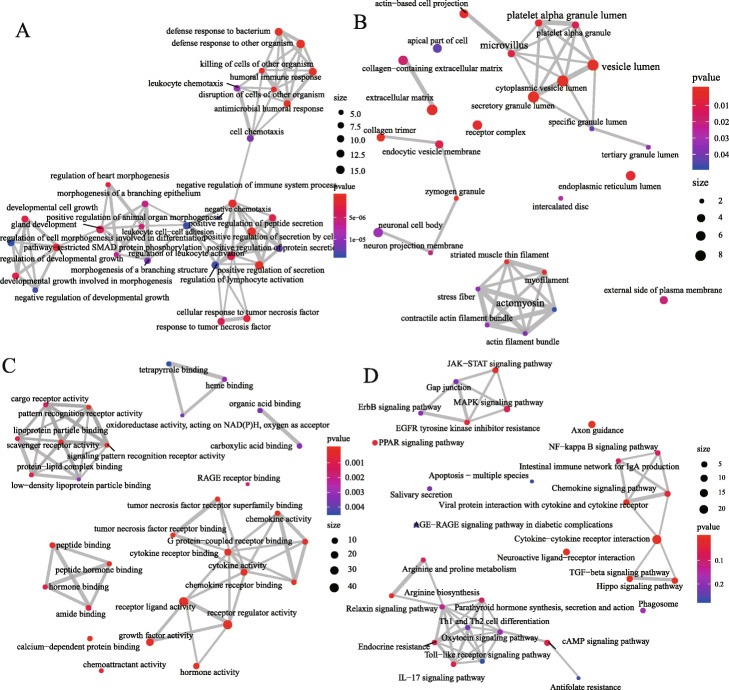
Functional-enrichment analysis of the 100 common differentially expressed genes. **a** Biological processes analysis, (**b**): Cell components analysis; (**c**): Molecular functions analysis; (**d**) Pathway analysis of the top 30 most important entries in the Kyoto Encyclopedia of Genes and Genomes. Significance gradually increases from blue to red; the thickness of the line represents the degree of correlation between the two points, and the size of the circle represents the number of genes enriched on the entry

The results of IRGs enrichment analysis were more common in the inflammatory pathway, including “positive regulation of secretion by cell,” “positive regulation of secretion,” “antimicrobial humoral response” and “defense response to bacterium” in biological processes. In the meantime, these genes participated in “secretory granule lumen,” “Cytoplasmic vesicle lumen,” and “vesicle lumen” in cell components; and played a main role in the regulation of various receptor ligands, cytokines, cytokine receptors, hormones, or chemokines in molecular functions. Also, these IRGs could be involved in the composition of signal pathways such as “Cytokine-cytokine receptor interaction,” “Axon guidance,” “TGF-beta signaling pathway,” “Viral protein interaction with cytokine and cytokine receptor,” and “Hippo signaling pathway” (Fig. 3). The above enriched items are all related to immunity or tumour, indicating that these 100 IRGs may play a role in regulating HCC by regulating some immunological process.

### Establishment and validation of a seven-gene prognostic signature based on the prognosis of HCC

**Fig. 4:**
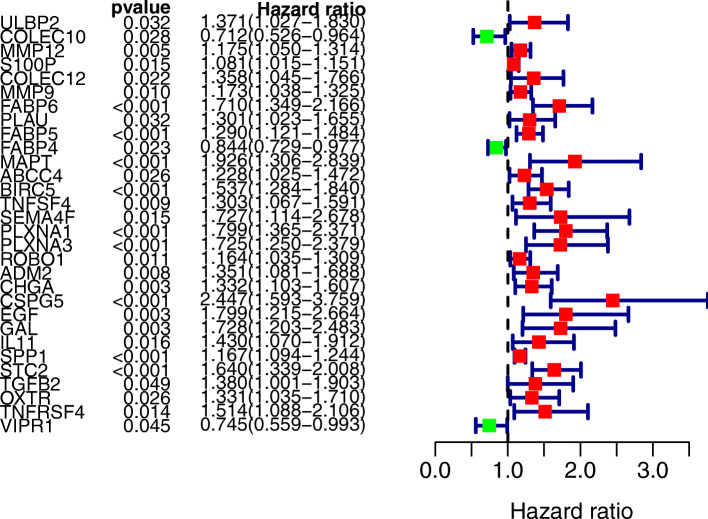
Forest plot of significant genes in univariate cox regression analysis (The red squares on the right side of the forest map indicate risk factors, and the green squares indicate protective factors)

We included a total of 343 patient cases (OS > 30 days) from TCGA in the survival analysis; OS was selected as the primary endpoint for this study. Applying the univariate Cox regression model (*P* < 0.05), we used the 100 IRGs to identify the DEGs associated with OS in HCC. We identified 30 OS-related DEGs, which were considered to be significant genes associated with HCC (Fig. 4).

**Fig. 5:**
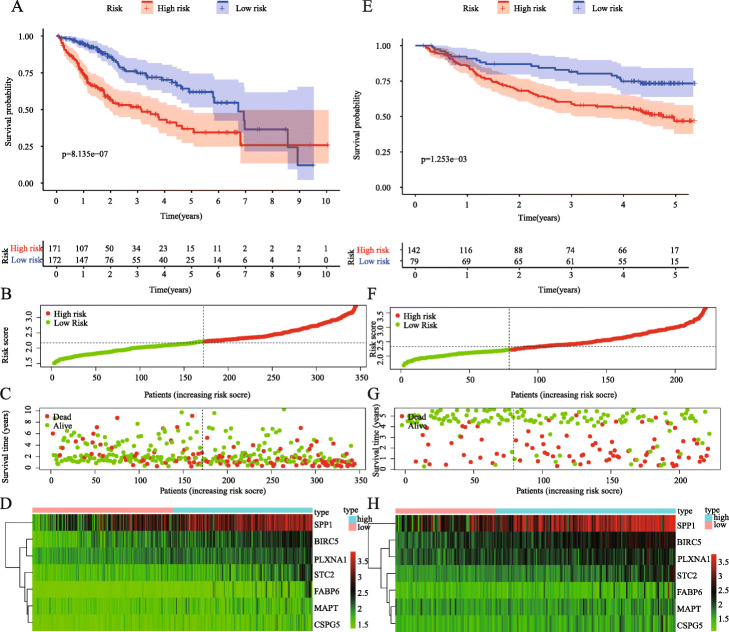
Construction of seven immune-related prognostic signatures for HCC. **a**: Kaplan-Meier curve for low- and high-risk populations in training group; (**b**): The distribution of risk score in patients in training group; (**c**): Survival status of patients with HCC in training group; (**d**): Heatmap of the expression levels of seven immune-related genes (IRGs) of patients in training group; (**e**): Kaplan-Meier curve for low- and high-risk populations in testing group; (F): The distribution of risk score in patients in testing group; (**g**): Survival status of patients with HCC in training group; (H): Heatmap of the expression levels of seven IRGs of patients in testing group

Using Lasso Cox multivariate analysis, we then developed an IPM based on seven genes: *FABP6*, *MAPT*, *BIRC5*, *PLXNA1*, *CSPG5*, *SPP1* and *STC2*. The hazard ratios of all these DEGs were > 1, meaning that all were considered oncogenes. According to the following formula:

$$ {\displaystyle \begin{array}{c} risk\ score=\left[(0.103)\times FABP6\  standardized\ expression\ value\right]+\\ {}\left[(0.0214)\times MAPT\ standardized\ expression\ value\right]+\\ {}\begin{array}{c}\left[(0.161)\times BICR5\  standardized\ expression\ value\right]\\ {}\left[(0.0421)\times PLXNA1\  standardized\ expression\ value\right]+\\ {}\begin{array}{c}\left[(0.244)\times CSPG5\  standardized\ expression\ value\right]+\\ {}\left[(0.0497)\times SPP1\  standardized\ expression\ value\right]+\\ {}\left[(0.174)\times STG2\  standardized\ expression\ value\right]\end{array}\end{array}\end{array}} $$

we calculated the risk score of each sample and then automatically divided all of the patients in TCGA into high- and low-risk groups according to median risk value. The K-M survival curve showed a significantly worse prognosis in the high-risk group (*P* = 8.135e− 07; Fig. 5a). The heatmap shows that as the risk score increases, the expression of IRGs gradually increases, thus indicating that high expression of these genes is a risk factor for HCC prognosis (Fig. 5b). In addition, the survival risks of these patients gradually increased as risk scores increased, and the number of survivors decreased significantly (Fig. 5c, d). Finally, we get the same results in the GSE14520 dataset, indicating that our model has a high degree of credibility (Fig. 5e-h).

**Fig. 6:**
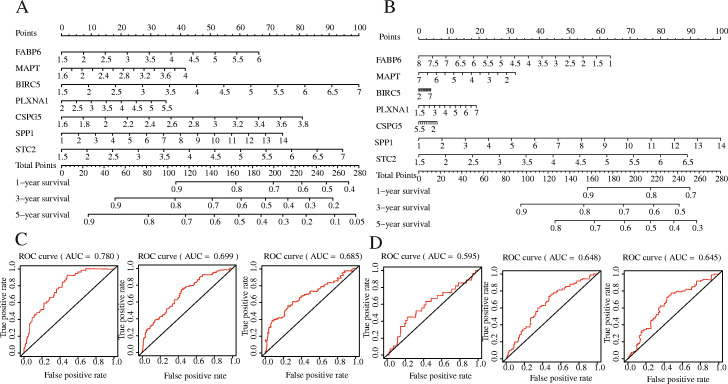
The establishment and verification of gene-related nomograms in the training (**a**, **c**) and verification (**b, d**) groups for predicting 1-year, 3-year, and 5-year survival rates of HCC patients

Meanwhile, we constructed a nomogram of the seven IRGs and evaluated the prognostic value of the seven-gene model based on the time-dependent ROC curve and the C-index value. The 1-, 3-, and 5-year risk prediction area under ROC curves (AUCs) for OS were 0.780, 0.699, and 0.685, respectively, and the C-index is 0.72, 95% [confidence interval (CI): 0.68, 0.77] and 0.62, 95% [CI: 0.57, 0.68], respectively. The above results indicated that the seven signatures performed well in predicting the OS of HCC, and we obtained the same results in testing set, too (Fig. 6).

### TF regulatory network

**Fig. 7:**
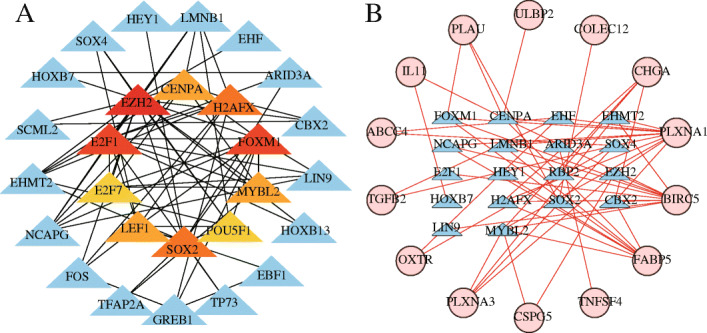
Protein–protein interaction network based on prognosis-related transcription factors (TFs) (**a**) and the main regulatory network between TFs and prognostic immune-related genes (IRGs) (**b**). In (**a**), center, the top 10 genes are sorted by the Degree criterion; the darker the color, the higher the ranking. In (B), the blue triangles represent TFs, the red circles represent differentially expressed prognostic IRGs, and the red connecting lines represent positive regulation

To explore the clinical significance of pivotal IRGs and the corresponding underlying molecular mechanisms, we examined the expression profiles of 318 TFs and found that 31 genes were differentially expressed between HCC and non-tumour HCC samples (|log FC| > 2, *P* < 0.05), and they were related to OS in HCC patients. Then, we established a regulatory network based on these 31 TFs and 9 IRGs that had proven significant in univariate analysis. Correlation coefficients > 0.4 and *P*-values ≤0.001 were set as screening criteria. The TF-based regulatory diagram in Fig. 7 clearly illustrates the regulatory relationship between these IRGs (Fig. 7).

### Evaluation of prognostic factors associated with OS in HCC

**Fig. 8:**
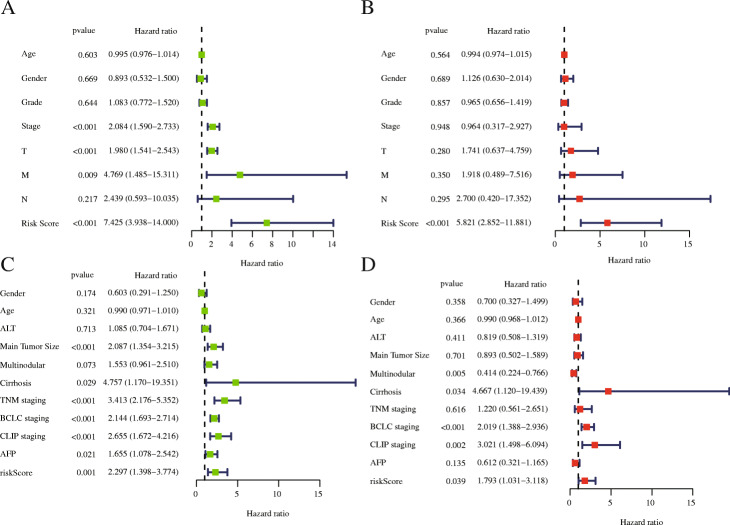
(**a**, **c**) Univariate and (**b**, **d**) multivariate Cox regression analysis of the correlation between risk score and clinical factors in training (**a**, **b**) and testing (**c**, **d**) group

We included 219 patients with complete clinical information in the TCGA-LIHC dataset. As important clinical indicators, gender, age, grade, and TNM staging were included in our study to identify prognostic factors. We used univariate and multivariate Cox regression analysis to determine prognostic factors associated with OS in HCC. Univariate analysis showed that risk score, TNM staging, T stage, and M stage were significantly correlated with OS (*P* < 0.05). Based on univariate-analysis results with *P* < 0.669, we further included these parameters in multivariate Cox regression analysis for analysis. Multivariate analysis showed that risk score (*P* < 0.001) was an independent risk factor (Fig. 8a, b), further demonstrating that our IPM’s impact on the patient’s prognosis is not disturbed by other clinical factors, and it is an independent prognostic factor of OS in HCC patients.

The clinical information of 242 HCC patients who meet the criteria in the GSE14520 dataset includes age, ALT (>/<=50 U/L), main tumour size (>/<=5 cm), multinodular cirrhosis, TNM staging, BCLC staging, CLIP staging and AFP (>/<=300 ng/ml) were included in the analysis. Univariate analysis showed that risk score, main tumour size, cirrhosis, TNM staging, BCLC staging, CLIP staging and AFP were related to OS; while multinodular, cirrhosis, BCLC staging, CLIP staging and risk score were independent prognostic risk factors in multivariate analysis (Fig. 8c, d).

### Construction and validation of a prognostic nomogram

**Fig. 9:**
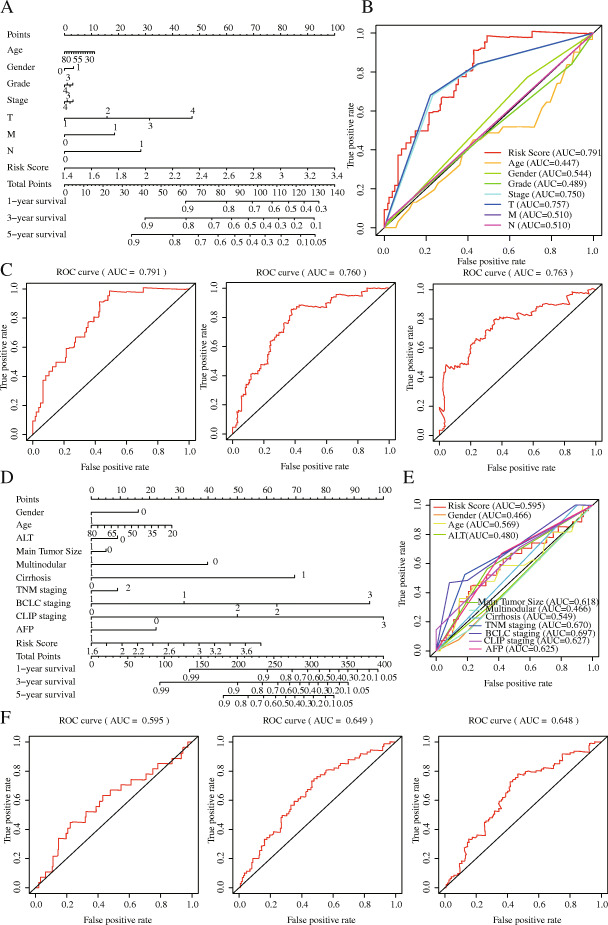
The establishment and verification of clinical-related nomograms in the training (**a**-**c**) and verification (**d**-**f**) groups for predicting 1-year, 3-year, and 5-year survival rates of HCC patients

**Fig. 10:**
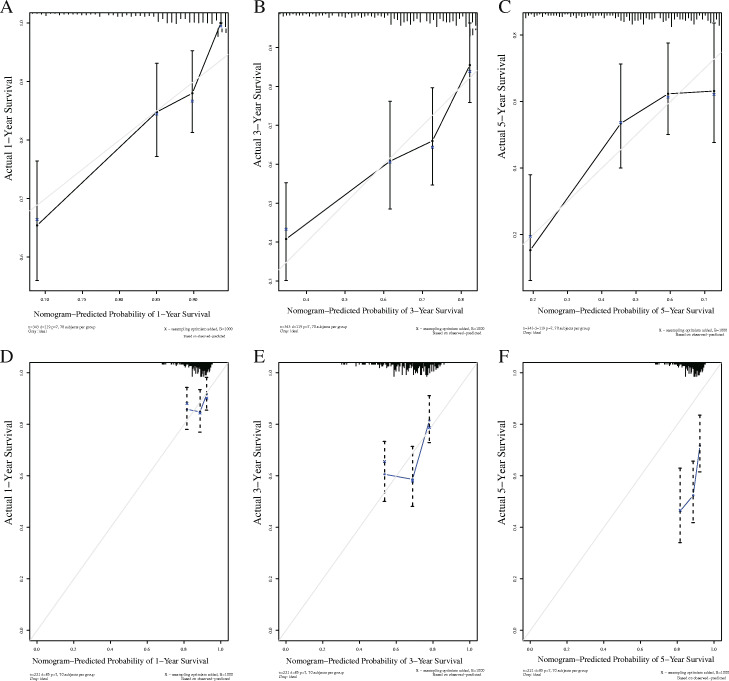
Calibration curve of nomogram in the training set and testing set. The X-axis is the predicted survival rate, and the Y-axis is the actual survival rate. **a, b, c**: 1-year, 3-years, and 5-year calibration curves in the training set; (**d**, **e**, **f**):1- year, 3- year and 5 -year calibration curves in the testing set

We used a stepwise Cox regression model to establish a prognostic nomogram based on the 219 eligible HCC patients with complete clinical information in the TCGA–LIHC dataset for predicting survival at 1, 3 and 5 years. Risk score, age, sex, TNM stage, T stage, N stage, and M stage were all nomogram parameters. The AUCs of OS at 1, 3 and 5 years were 0.791, 0.760 and 0.793, respectively. The C-index was values were 0.78 (95% CI: 0.72, 0.84) and 0.73 and (95% CI: 0.68, 0.78) in the training and testing groups, respectively. The results of the clinical factors showed that the AUC values of T stage, TNM stage, and risk score were the highest at 0.757, 0.750, and 0.791, respectively, which suggested that the IPM had moderate prognostic performance (Fig. 9). The calibration curve further showed that the nomogram performed well in predicting the OS of HCC patients in the training group. However, the difference between the predicted survival rate and the actual survival rate in the calibration curve of the testing group was large, suggesting that the performance of the prognostic model may need to be further verified (Fig. 10).

### Gene set enrichment analysis

**Fig. 11:**
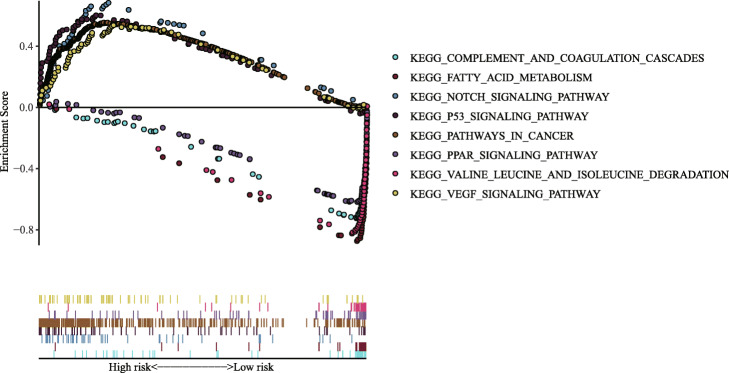
Representative pathways of significant enrichment in the model by Gene set enrichment analysis (high-risk group above, low-risk group below)

We performed GSEA in the training group to identify the differences between the high-risk and low-risk groups. Among them, the high-risk group had 41 significantly enriched pathways, and the low-risk group had 12 significantly enriched pathways. The enrichment pathways in high-risk groups are mostly related to tumours (bladder cancer, small cell lung cancer, non-small cell lung cancer, pancreatic cancer and colorectal cancer) or tumour-related pathways (“p53 signaling pathway”, “nucleotide-binding oligomerization domain (NOD) -like receptor signaling pathway”, “Notch signaling pathway”, “VEGF signaling pathway” and “Pathways in cancer”), and metabolic or metabolic disease-related pathways (pyrimidine metabolism, purine metabolism and N-Glycan biosynthesis); the enrichment pathways in the low-risk group are mostly related to metabolism (fatty acid metabolism, valine leucine and isoleucine degradation, drug metabolism cytochrome P450 and tryptophan metabolism), complement and coagulation cascades and the PPAR string pathway (Fig. 11).

### The correlation between clinical factors and gene signature in IPM

We analysed the relationship between genetic characteristics and clinical parameters (Table 2) in training group. Compared with patients with Stage I/II, G1/G2, and T1/T2 in HCC, patients with Stage III/IV, G3/G4, and T3/T4 have higher levels of *BICR5* gene expression and risk score. Female has higher expression level of *PLXNA1* than male, while G3/G4 and stage III/IV have higher expression level of *PLXNA1* gene. Those of patients in Stage III and IV were higher in *SPP1* expression than those of patients in Stage I and II, a difference that was statistically significant. Patients in stage N0 had higher expression of *FABP6* and *MAPT* than patients in stage N1, probably due to the large difference in the number of samples between the two groups. In terms of survival time, patients with HCC in T1/T2 and Stage I/II were significantly higher than those with the disease in T3/T4 and stage III/IV.

**Table 2:** The relationship between clinical factors and risk scores or the expression of seven prognostic related immune genes in hepatocellular carcinoma

ID (t/P)	Age	Gender	Grade	Stage	T	M	N
Futime	1.613 (0.110)	0.772 (0.442)	0.59 (0.556)	**2.533 (0.012)**	**2.572 (0.011)**	1.716 (0.212)	0.661 (0.570)
FABP6	0.727 (0.469)	−0.295 (0.768)	**−1.975 (0.050)**	− 1.163 (0.247)	− 1.23 (0.222)	− 0.363 (0.750)	**2.93 (0.017)**
MAPT	0.599 (0.550)	1.337 (0.184)	0.556 (0.579)	−1.101 (0.273)	−1.373 (0.173)	0.935 (0.439)	**11.791 (4.946e-14)**
BIRC5	−1.645 (0.102)	0.294 (0.769)	**−3.054 (0.003)**	**−2.601 (0.011)**	**−2.563 (0.012)**	2.133 (0.124)	−1.121 (0.374)
PLXNA1	−0.668 (0.506)	**2.563 (0.012)**	**−2.456 (0.015)**	**−2.198 (0.030)**	−1.61 (0.110)	1.044 (0.400)	−3.213 (0.082)
CSPG5	−0.632 (0.529)	−0.448 (0.655)	−1.902 (0.059)	−1.288 (0.201)	− 1.383 (0.170)	2.511 (0.055)	0.868 (0.466)
SPP1	0.514 (0.608)	−1.584 (0.116)	−1.325 (0.187)	**−2.061 (0.042)**	− 1.843 (0.068)	−0.535 (0.646)	−0.174 (0.878)
STC2	1.179 (0.242)	0.715 (0.476)	−1.356 (0.177)	−1.599 (0.113)	− 1.504 (0.136)	−0.943 (0.444)	−0.177 (0.875)
Risk Score	−0.09 (0.929)	−0.146 (0.884)	**−3.027 (0.003)**	**−2.955 (0.004)**	**−2.819 (0.006)**	− 0.362 (0.750)	− 1.56 (0.226)

Note: *t* t value of student’s t test, *P*: *P*-value of student’s t test

### Association between the degree of immune infiltration and risk score

**Fig. 12:**
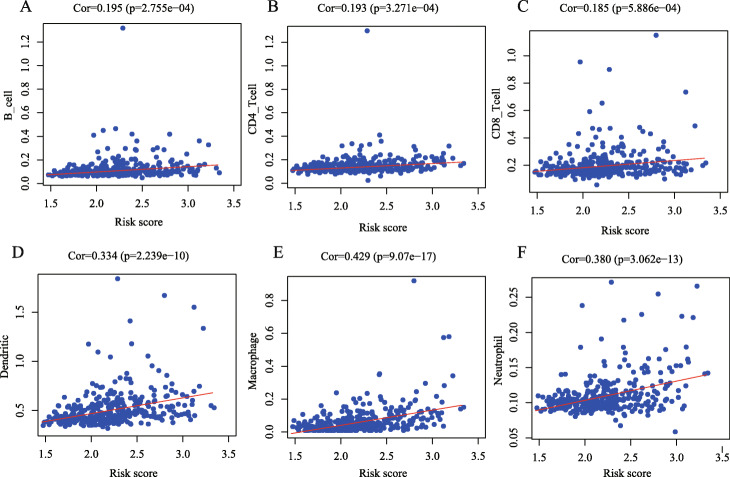
Pearson correlation analysis between risk score and infiltration abundances of six types of immune cells. **a** B cells, (**b**) Cluster of Differentiation 4–positive (CD4+) T cells, (**c**) CD8+ T cells, (**d**) neutrophils, (**e**) macrophages, and (**f**) dendritic cells

To further study whether risk score in this IPM could affect the abundance of immune cells in the TME, we performed a correlation analysis between six types of common immune cells and risk score. In Fig. 12 we can see that the correlation coefficients of risk score and neutrophils, TAMs and dendritic are all above 0.2; B cells, CD4^+^ T cells and CD8^+^ T cells and risk scores is less than 0.2, but it is relatively close to 0.2. The results showed that all immune cells were positively correlated with risk score to a statistically significant degree (Fig. 12; *P* < 0.05), which implying that the higher the degree of immune infiltration, the worse the prognosis of the patient.

### Verification of hub genes using online website

**Fig. 13:**
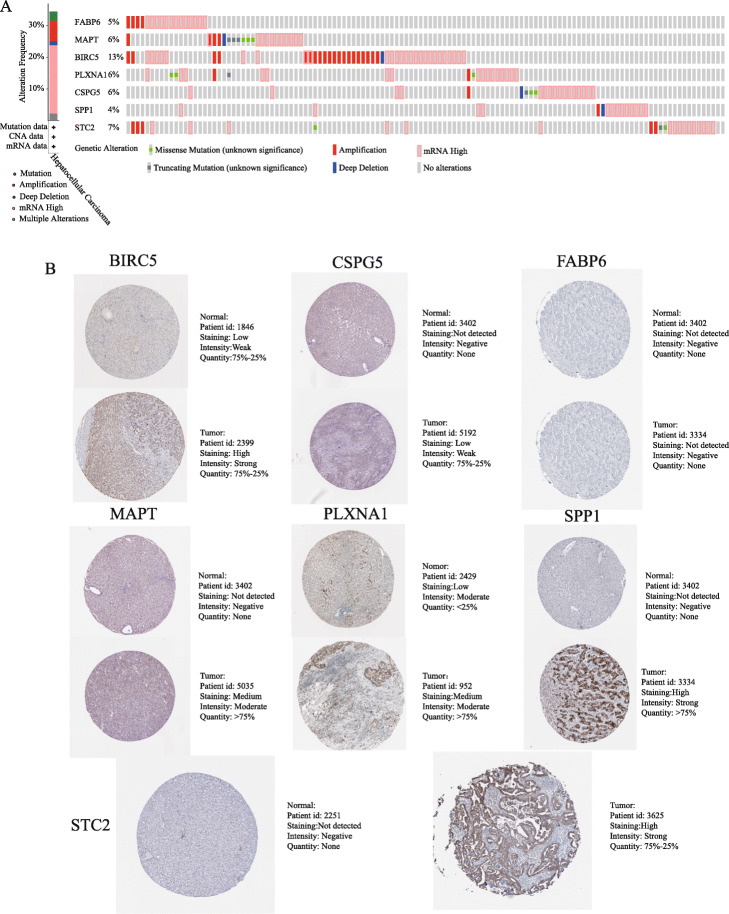
Genetic alterations landscape (**a**) and expression in the translational level (**b**) of the seven-prognostic immune-related genes in hepatocellular carcinoma

Based on the above analysis results, we can see that the model has a high clinical application value, so we conducted a study of the molecular characteristics of the IRGs in the IPM. We analysed the genetic-variation results of the genes *FABP6*, *MAPT*, *BIRC5*, *PLXNA1*, *CSPG5*, *SPP1* and *STC2*. Of the 349 patients included in the cBioPortal, 120 (34.38%) showed genetic changes in these seven genes. With mRNA high (21.49%) was the most common genetic variation, amplification (6.5%) and missense mutation (1.69%) being the next most common (Fig. 13a). We further analysed the differences in the expression of these genes at the protein level. As shown in Fig. 11b, except for *FABP6*, the other genes were all highly expressed in HCC tissues.

**Fig. 14:**
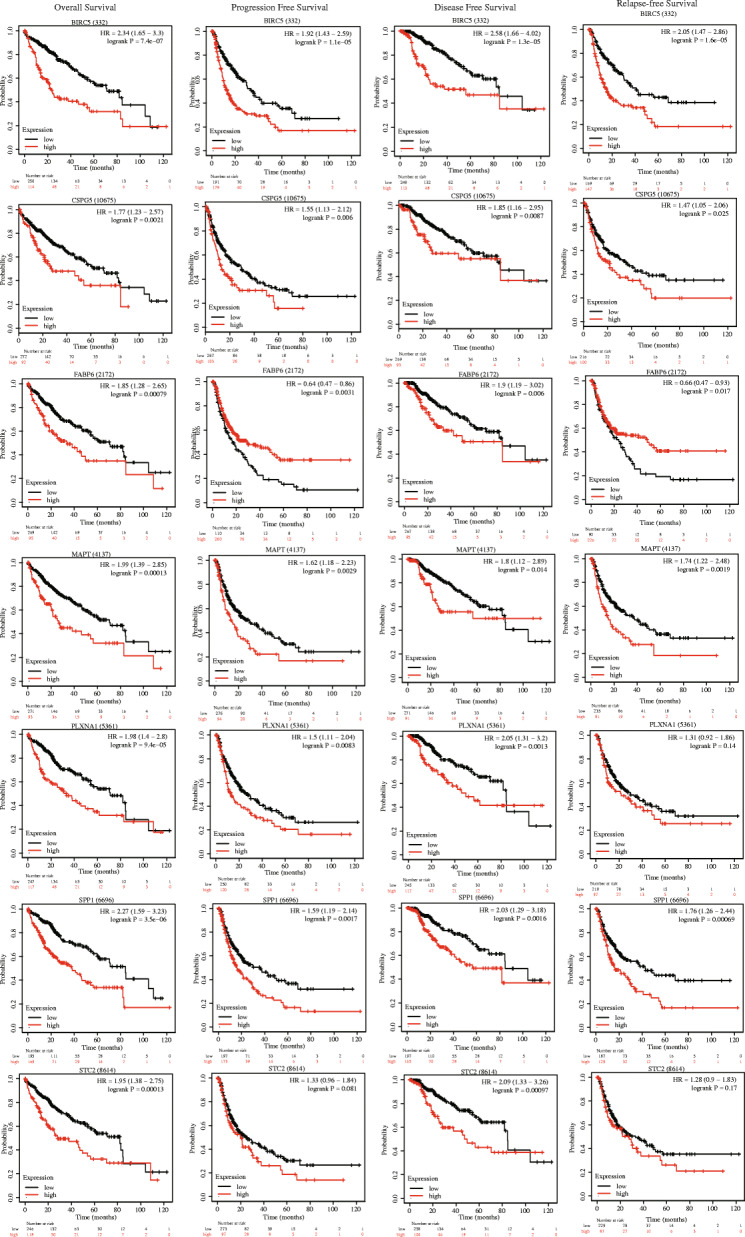
Overall survival, progression-free survival, disease-free survival, and relapse-free survival Kaplan-Meier curves of seven prognosis-related IRGs (From top to bottom: *BIRC5*, *CSPG5*, *FABP6*, *MAPT*, *PLXNA1*, *SPP1*, *STC2*). Black curve represents low expression; red curve represents high expression

To confirm that multi-gene signatures have better prognostic performance than a single-gene signature, we performed a K-M survival analysis of the seven IRGs in this IPM (Fig. 14). Almost all of the *P*-values were < 0.05, and the c-indexes in ROC curves were lower than in IPM, further proving the importance of these seven IRGs. Since these seven IRGs were found to be oncogenes, the high expression of genes is often associated with poor prognosis, which is basically consistent with the conclusions we have obtained before. However, patients with high expression of *FABP6* had longer PFS and RFS. Our conclusions regarding *FABP6* was unclear, and further research might be needed to verify them. In short, the abnormal stomatic mutations, expression and survival differences of these seven IRGs in HCC may help explain their important application value.

## Discussion

Components of the TME, the environment in which tumour cells grow, include inflammatory cells, fibroblasts, myofibroblasts, neuroendocrine cells, adipocytes, and extracellular matrix [51]. The TME is inseparable from the growth, invasion, metastasis, and prognosis of tumour cells [45]. Unlike cancer cell genes and epigenetic mechanisms, the matrix population in the TME is relatively stable genetically; therefore, having potential therapeutic value. A growing number of studies have begun to focus on the regulatory mechanism of the TME on HCC [45]. Although major results have been achieved, identifying suitable immunotherapeutic targets in complex TME components requires the joint efforts of multiple research teams. The application of molecular prognostic models and the identification of target genes can be described as molecular therapeutics that may provide effective approaches in the future [3]. Fortunately, the open source TCGA and GEO databases have accumulated an abundance of genomic information, providing a simpler and more reliable way to predict prognosis in cancer. Our study intended to identify IRGs from the TCGA and ImmPort databases that were significant to HCC prognosis, and further verified the results in the GSE14520 dataset. The identification of these IRGs and the construction of the IPM might provide new ideas for immunotherapy in HCC. Finding and developing novel targets with potential value for immunotherapy is very important; after experimental verification and clinical trials, it can strengthen current immunotherapeutic regimes.

In the current study, we obtained 2068 HCC-related DEGs from TCGA and finally retained 100 IRGs that also existed in the GEO database for model construction. After identifying the common DEGs in the training set and testing set, we used only the IRGs to construct a Cox model for predicting OS. Incorporating clinical factors and genome information into Lasso regression analysis is indeed a better modelling method, and it is also the current trend of machine learning. However, doing this makes it difficult for us to externally verify the model because the clinical information contained in each data set is different, and there are certain limitations in selecting limited clinical factors. In addition, each type of tumour has different susceptibility factors and important indicators. Taking lung cancer as an example, attention needs to be paid to the influence of radon, asbestos, second-hand smoke and other factors, while in HCC, attention needs to be paid to hepatitis history, liver cirrhosis and AFP. Therefore, these models may not be generalizable to other types of cancer. Konstantina Kourou [52] summarized and analysed a number of studies that incorporated clinical factors and genomic information into models, but their research lacks external verification or testing of the predictive performance of the models.

In this case, other clinical information was omitted since we sought to create a simple but powerful model that could be externally verified by various data sets. Then, we evaluated the model, including the AUC curve and C-index, as well as the calibration plot, and made comparisons with the prognostic model constructed based on the risk score and other clinical factors to prove the advantages of the proposed modelling scheme. With the development of high-throughput sequencing technology and the generalization of clinical genetic testing, the use of genomic information to construct a predictive model for the simple prognostic analysis of patients will bring certain convenience to the clinic. Perhaps with the continuous improvement of various public databases, the joint modelling and analysis of clinical information and multiomics data will also become a trend.

Further gene function analysis results showed that all of the IRGs were mainly enriched in the positive regulation of secretion by cells, secretory granule lumen, receptor ligand activity, and cytokine-cytokine receptor interaction (Fig. 3). Specifically, these IRGs are mainly involved in various immune regulation processes (such as positive regulation of secretion by cells, positive regulation of secretion, antimicrobial humoral response, defence response to bacteria and humoral immune response) and take part in the composition of the secreted granule lumen, cytoplasmic vesicle lumen, and vesicle lumen; these IRGs regulate various receptors, ligands, growth factors, cytokines, and chemokines. In addition, they were mainly enriched in cytokine-cytokine receptor interactions, axon guidance, the TGF-beta signalling pathway, viral protein interactions with cytokines and cytokine receptors, and the Hippo signaling pathway. Most of the above items are related to immunity and inflammation, and the rest are classic signalling pathways in tumours. Jian Chen et al. reported that dysregulation of the TGF-beta signalling pathway plays a key role in immune regulation, inflammation and fibrogenesis in HCC [53]. Disorders of the Hippo signalling pathway are present in various tumours, including liver cancer [54], breast cancer [55] and lung cancer [56]. At present, immune checkpoint inhibitors can greatly improve the prognosis of HCC. However, the specific mechanism of the immune system affecting HCC are unclear, and further experiments is needed to confirm our conclusion.

In our research, we reached a nearly consistent conclusion with other researcher’s prognostic models: there were significant differences in OS between the high-risk and low-risk groups, the prognosis of the high-risk group was worse (Fig. 5a, *p* = 8.135 × 10^− 7^), and the same conclusion was reached in the testing set (Fig. 5a, *p* = 1.2535 × 10^− 3^). The patient’s risk score for HCC progressively increases as the expression levels of the genes in the risk signature increase, and the prognosis of HCC worsens as the risk score increases. More importantly, we constructed a nomogram based on these seven IRGs to quantitatively analyse the prognosis of HCC patients. The AUCs for 1-, 3-, and 5-year OS were 0.780, 0.699 and 0.685, respectively, and the C-index was 0.72 (95% CI: 0.68–0.77). HCC is a highly heterogeneous disease, and its prognosis is affected by many factors. We only included and analysed genes related to immunity and ignored the influence of other factors on HCC. Hence, our model does not show a high prognostic performance in predicting the long-term survival rate of patients, which is also one of the inherent defects of the model. We further analysed the risk score and clinically related factors in univariate and multivariate analyses and found that the risk score was associated with higher grade (*P* = 0.003) and stage III/IV disease (*P* = 0.004), which indicated that our prognostic model was more significant in advanced HCC patients. We believe that genetic detection should not be considered independently of individual characteristics. Therefore, we also constructed a nomogram combining the risk score and clinical factors, which can easily predict the 1-year, 3-year and 5-year OS of patients. It should be noted that the AUC values were all higher than 0.7. Compared with other clinical factors, the AUC value of the nomogram corresponding to risk score was the highest (AUC = 0.791), and the C-index was 0.78 (95% CI: 0.72–0.84). In addition, when we analysed the risk score combined with clinical factors, the C-index of the test dataset was 0.73 (95% CI: 0.67–0.78), indicating that our IPM has a modest prognostic performance in the test dataset. In the GSE14520 dataset, a series of test results were basically consistent with those in the TCGA dataset. Although the AUC values reached above 0.5 (Fig. 6), the same effect as that in the training set was not achieved, which may be because the samples in the GSE14520 dataset were from China. Generally, the model constructed in this study has certain advantages in the quantitative prediction of patient prognosis and adjustment of the treatment plan.

Our research results showed that the risk score is the only meaningful indicator in multiple analyses, which indicates that the risk score may have a better predictive ability for the OS of HCC. However, the standard error of an estimate does not tell us about the estimate’s contribution to a prediction model. Nonsignificant coefficients can still have very high predictive power, and vice versa. In addition, a significant covariate doesn’t imply a reliable estimation of survival; thus, we still need to assess the model. Next, we performed the same analysis in the test dataset and obtained the same conclusion. In addition, we evaluated our model with the AUC curve, C-index and calibration curve, which suggested that our model has good prognostic performance. To further verify the prognostic performance of the risk score, we compared the AUC value of the model constructed by the risk score with that of the model constructed by other clinical factors (Fig. 9b, e), and the results indicated that the risk score may be a good predictor of HCC survival. In addition, compared with a single gene, prognostic models based on multiple genes can better analyse the prognosis of patients. To develop a simple and effective method for evaluating the prognosis of HCC patients and find potential immunotherapy targets, we established a prognostic model based on the seven IRGs. Of course, ours is not the first IPM for HCC. Wen-jie Wang et al. constructed a prognostic model of 16 IRGs and a ceRNA network to predict the prognosis of HCC [57]; Junyu Long et.al developed a HCC immune prognostic model related to *TP53* [28]; and Dengchuan Wang et al. reported a four-gene signature prognostic model related to immune infiltration through coexpression analysis [57]. Recently, an increasing number of researchers have begun to recognize the significance of the TME in HCC, and IPMs have also received extensive attention. Compared with other prognostic models, our IPM has the following advantages. (1) We have not only established a seven-gene prognostic model of IRGs but also showed that the model can be independent of other clinical factors and is positively correlated with the degree of immune infiltration, which can provide valuable prognostic information for optimizing the individual treatment of HCC patients. Additionally, we constructed a gene nomogram and clinically related nomogram to quantitatively evaluate the 1-, 3-, and 5-year OS of patients. (2) We constructed a TF regulatory network, performed GSEA and analysed the possible mechanisms of the IRGs in the IPM related to HCC tumour infiltration, which can contribute to exploring the immunotherapy mechanism of HCC. (3) We performed gene mutation analysis and protein expression level analysis on the genes in this IPM, and also analysed the survival differences between patients with high and low expression levels of the IRGs. The conclusions obtained further confirmed the potential of IRGs in the model as a prognostic marker of HCC.

The signatures in this IPM have good prognosis performance, which could be potential prognosis and therapeutic targets for HCC. *BIRC5*, commonly known as *Survivin*, is the most effective molecule in inhibitor-of-apoptosis [58]. Experimental investigation showed that *BIRC5* can promote the expression of *VEGF*, which in turn promotes angiogenesis in the tumour stromal [59]. *PLXNA1* (Plexin-A1) is expressed in DC and participates in the interaction between T cells and DC, and may be involved in regulating the rearrangement of the cytoskeleton during the interaction between T cells and DC [60]. *CSPG5* is only expressed in the human brain, and a study showed that it has a new function that binds to *ERBB3* tyrosine kinase [61], and the *ERBB3* somatic mutation is a potential tumour driver [62]. However, few studies have focused on its relevance to HCC immunotherapy. Ying Zhu et al. found that *SPP1* can activate the CSF1-CSF1R pathway in tumour-associated TAMs and promote the expression of PD-L1 in HCC, and there is a positive correlation between *SPP1* and PD-L1, TAM expression. On the other hand, *SPP1* can induce endothelial cells and upregulate *VEGF*-induced migration of endothelial cells, having a synergistic effect with *VEGF* in tumour angiogenesis [63]. *MAPT* is mainly expressed in nerve cells, and more commonly studied in geriatric diseases such as various neurodegenerative diseases including Alzheimer’s disease [64]. Previous investigation identified that *MAPT* is overexpressed in certain cancers, and participate in the resistance of various tumours to taxane drugs [65], and its specific mechanism of action still needs further study. *FABP6* is involved in the bile acid metabolic process and is related to the bile acid intestinal circulation [66]. *STC* plays a vital role in tumour growth, invasion, apoptosis and metastasis, and promotes local angiogenesis through the *VEGF/VEGFR2* signalling pathway [67]. Hongwei Cheng et al. showed that the high expression of *STC2* is related to the poor prognosis of HCC [68]. To further confirm the application value of these IRGs in HCC, we analysed the survival rates of groups with high- and low- expression levels of these seven genes and evaluated whether there was significance in patients’ OS, DSS, PFS, and RFS rates. The results showed that there were some contradictions in the survival analysis of *FABP6*. Also, the results of immunohistochemistry in the HPA database showed that except *FABP6*, the protein levels of other IRGs were significantly different between HCC tissues and normal liver tissues, and they were highly expressed in HCC, which was consistent with our conclusion. At present, *FABP6* has not been reported in immunotherapy of HCC, which may be potential therapeutic targets for HCC. We then used the seven IRGs from the cBioPortal to obtain information about genetic mutations. The mutation rates of these seven IRGs are more than 4%, which may be useful for clinical research in the future. Collectively, these findings indicated that the seven IRGs have the potential to predict the prognosis of HCC.

To explore the potential molecular mechanisms associated with these IRGs, we constructed a TF-mediated regulatory network to screen out important TFs that might regulate identified the hub IRGs. *BIRC5, PLXNA1* and *CSPG5* were the core IRGs in this network; all three were positively regulated by 13 core TFs, of which *EZH2* could positively regulate the expression of *BIRC5, PLXNA1* and *CSPG5*. *EZH2*, the hub TF in our PPI analysis (Fig. 6a), is shown in the network diagram (based on the degree ranking criterion). An accumulating number of studies show that *EZH2* is closely associated with of epigenetics [69], immunity [70], metastasis [71], angiogenesis [72] and apoptosis [73]. In addition, our GSEA results showed that these seven IRGs play an important role in tumour regulation in the high-risk and low-risk groups through immune metabolic pathways. Gaia Giannone demonstrated that immune metabolic disorders play an important role in acquired resistance to the TME and immune checkpoint inhibitors [74]. At present, the *VEGF* signalling pathway is widely used in the immunotherapy of HCC [75] and may be involved in cell proliferation, growth and apoptosis processes as well as the regulation of the *PPAR* and *TP53* signalling pathways [76]. NOD-like receptor X1 can induce HCC cell apoptosis by regulating the *PI3K-AKT* signaling pathway [77]. The inhibition or promotion of the Notch signalling pathway in different tumours depends on the TME. The cross-talk between the Notch signalling pathway and *p53* gene plays an important role in HCC and may be a potential target for HCC treatment [78]. Of particular note, based on the above studies, we found that *EZH2* and *BIRC5* can inhibit HCC cell apoptosis and are closely related to *VEGF*-mediated angiogenesis. Interestingly, in the regulatory network of TFs, *EZH2* positively regulated *BIRC5*, with a correlation coefficient of 0.72 (*p* = 3.76 × 10^− 57^). *STG* and *SPP1* are associated with the *VEGF* signalling pathway, *PLXNA1* and *SPP1* are associated with DCs or TAMs; *CSPG5* is associated with common somatic mutation sites. The application values of *MAPT* and *FABP6* in HCC need further experimental confirmation. In this case, we boldly speculate that *EZH2* may mediate the angiogenesis of the *VEGF* signalling pathway through regulating the expression of the seven IRGs, which may be the possible mechanism of this predictive model related to immune infiltration in high-risk patients. In low-risk patients, we found that the mechanism of these seven IRGs related to the immune infiltration of HCC is related to metabolism. However, the specific mechanism remains to be further explored. The combination of antiangiogenic drugs and tumour immunotherapy will show great prospects in the near future. However, further insights by validation with immunohistochemistry analysis are needed to understand whether the *VEGF* signaling pathway is linked to high-risk groups.

To further assess the immune microenvironment of HCC, we also analysed the correlation between risk score and the following six types of immune cells: B cells, CD4^+^ T cells, CD8^+^ T cells, neutrophils, TAMs, and DCs. The results showed that for these six cell types, the degree of immune infiltration was positively correlated with the risk score, and the correlations between all immune cells and the risk score were statistically significant (*P* < 0.05). These results indicated that these cells have a high level of immune infiltration in high-risk patients. TAMs are phagocytes, which are the body’s first line of defence against external threats; they can produce proinflammatory responses to pathogens and repair damaged tissues. However, cytokines and chemokines expressed by TAMs can inhibit antitumour immunity and promote tumour progression [79]. The expression of M1 macrophages in HCC can promote tumour formation by promoting the expression of PD-L1, and their infiltration degree is positively correlated with the expression of PD-L1. On the other hand, Ying Zhu et al. found that there was a positive correlation between the expression of *SPP1* and PD-L1 and the infiltration of TAMs in HCC tissues, which played an important role in the immune microenvironment of HCC [80]. All these results suggested that our high-risk patients may benefit from PD-L1 treatment. Li Li et al. [81] illustrated that the CXCR2-CXCL1 axis can regulate neutrophil infiltration in HCC; this axis is an independent prognostic factor for HCC and may be a potential target for anti-HCC therapy. Overexpression of CXCL5 is associated with neutrophil infiltration and poor prognosis of HCC [82]. Wei Y et al. showed that the depletion of B cells can prevent the production of TAMs and increase the antitumour T cell response to inhibit the growth of HCC [83]. Several studies indicated that high infiltration levels of immunosuppressive TAMs and regulatory T cells are associated with reduced OS and can increase the aggressiveness of HCC [84, 85]. Research by Zhou ZJ et al. [86] illustrated that the high infiltration level of plasmacytoid DCs is related to the poor prognosis of HCC, and plasmacytoid DC infiltration in HCC can promote tumour progression by promoting the immunosuppression of CD4^+^ type 1 T regulatory (Tr1) cells [87], which is basically consistent with our research conclusions. The occurrence and development of HCC involve interactions between various immune cells, and participates in the regulation of HCC immunotherapy through a complex mechanism. Our IPM may be a predictor of increased infiltration of HCC immune cells. However, the correlation coefficient between the infiltration abundance of CD4^+^ T cells and CD8^+^ T cells and the risk score was less than 0.4. In addition, studies have shown that CD4^+^ T cells and CD8^+^ T cells can suppress the occurrence and proliferation of HCC due to their antitumour immune response [88]; however, another study indicated that the proportion and absolute number of CD4^+^ T cells in the area surrounding HCC tumour tissue increases significantly and could promote the progression of HCC [89]. Shinji Itoh et al. showed that the presence of CD8^+^T cells is associated with longer OS [90]. Although our conclusion is basically consistent with that of previous studies in terms of the immune infiltration of HCC, the differences we found were not was not related to any biologically relevant levels that should be interpreted outside this analysis in the future. Overall, this study can provide direction and guidance for the mechanism of immune cells in HCC, but the specific mechanism remains to be elucidated by further basic research.

We systematically and comprehensively analysed the application value of our IPM for HCC, which could provide new insights for the treatment of this disease. However, the current study still has certain limitations. The IPM constructed based on the TCGA database has the best predictive performance in the training set. In the testing set, the risk score is not the best indicator to predict the OS of HCC. Limited by clinical factors in the testing group, our model still needs to be further validated in other datasets. Although we increased the reliability of our conclusions by combining multiple datasets, evaluating various aspects of the IPM, and verifying our results using the GSE14520 database. Most of the studies are plagued by a lack of validation in vitro and in vivo validation experiments, and further evidence provided by a well-designed clinical study is needed. It is also essential to use relevant basic experiments to further explain the mechanism of HCC immunotherapy. On the other hand, we have studied six major immune cells, the correlation between immune cells and the risk score was weak, and the association between more immune cell subtypes and HCC is still unclear. Therefore, we suggest that further individual studies and discussions should be conducted in the future. We believe that the era of HCC immunotherapy will soon be realized, and we look forward to it.

## Conclusion

To sum up, we constructed an IPM with seven prognostic IRGs by combining different data types from multiple databases. Individuals with HCC were automatically classified into high-risk and low-risk groups based on their risk scores, with gene expression as an independent variable. In addition, we established gene-related and clinical factor-related nomograms to facilitate more-comprehensive prognostic assessments of HCC patients. Finally, the results of the association between infiltration abundance of common immune cells in the TME and risk score showed that our IPM could predict the TME to a certain extent. This model will be a reliable tool for predicting prognosis in HCC by combining genomic characteristics, immune infiltration abundance, and clinical factors.

## Data Availability

The datasets for this study can be found in TCGA [https://portal.gdc.cancer.gov/] and GEO databases [https://www.ncbi.nlm.nih.gov/geo/].
