# Correction: A prognostic model based on seven immune-related genes predicts the overall survival of patients with hepatocellular carcinoma

**DOI:** 10.1186/s13040-023-00347-9

**Published:** 2023-10-26

**Authors:** Qian Yan, Wenjiang Zheng, Boqing Wang, Baoqian Ye, Huiyan Luo, Xinqian Yang, Ping Zhang, Xiongwen Wang

**Affiliations:** 1 The First Clinical Medical School, Guangzhou University of Chinese Medicine, Guangzhou, China; 2 Department of Oncology, The First Affiliated Hospital, Guangzhou University of Chinese Medicine, Guangzhou, China

**Correction: BioData Mining 14, 29 (2021)**

**https://doi.org/10.1186/s13040-021-00261-y**

Following publication of the original article [1], the authors found that the pathology images of normal liver tissue and liver cancer tissue of FABP6 in Fig. 13B were duplicated. The pathological images of normal FABP6 and liver cancer patients were both sourced from public databases (The Human Protein Atlas, https://www.proteinatlas.org/). Therefore, the results in this article are reliable. To make this research repeatable, the following has provided a download link for pathological images.

The patient ID of normal liver tissue is 3402. link:

https://www.proteinatlas.org/ENSG00000170231-FABP6/tissue/liver#img;
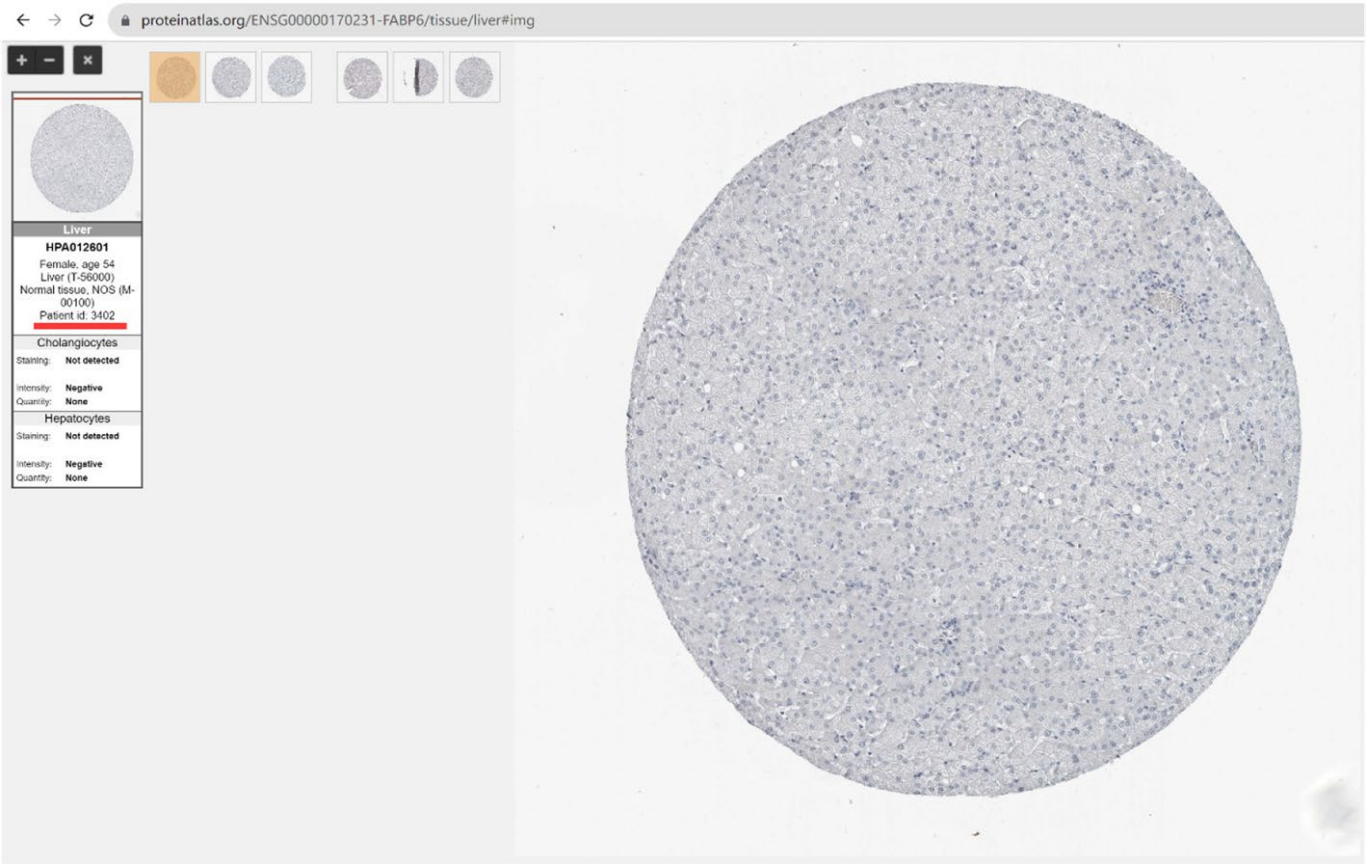


**FABP6 normal**

The liver cancer patient ID is 3334, link:

https://www.proteinatlas.org/ENSG00000170231-FABP6/pathology/liver+cancer#img.
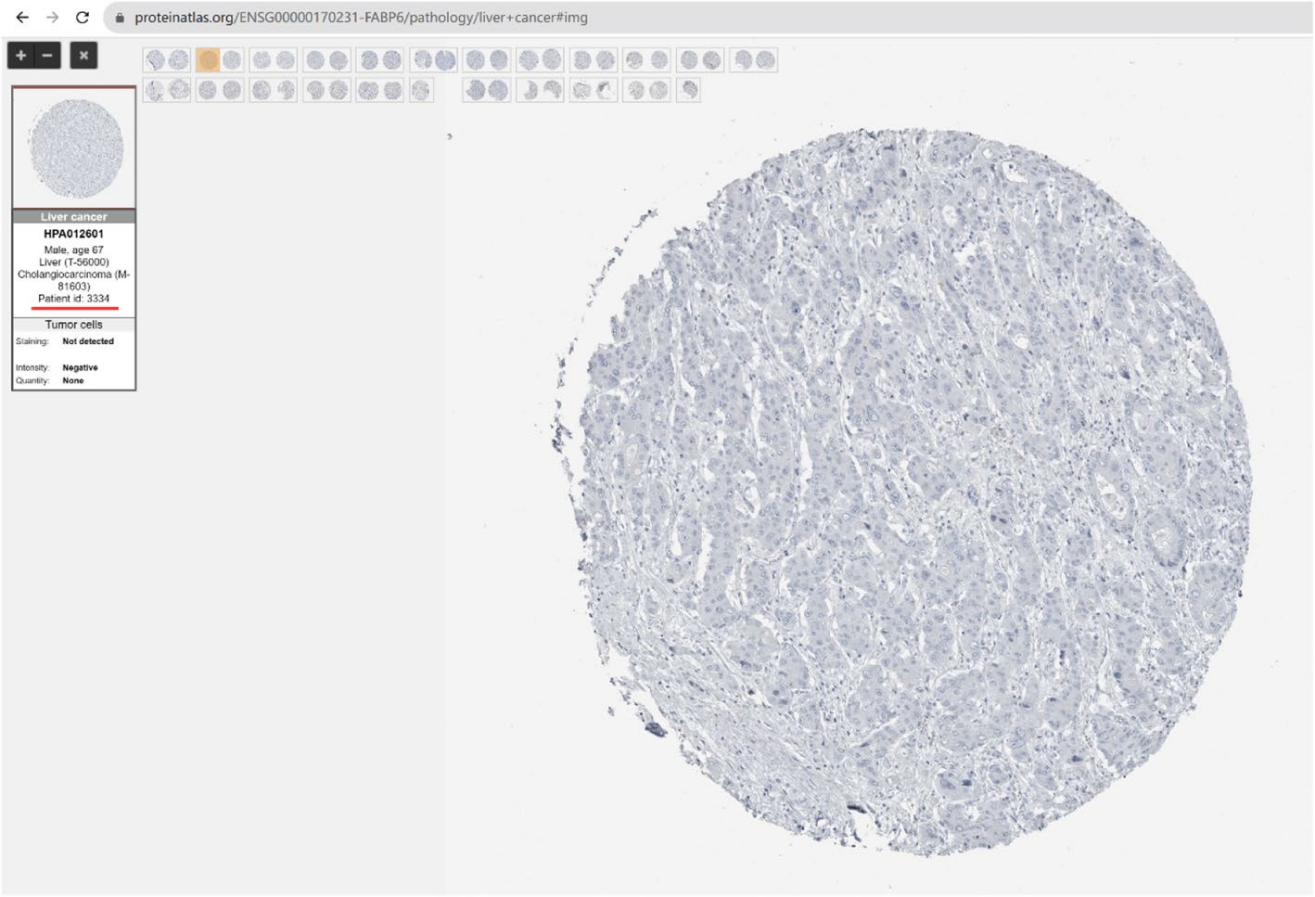


**FABP6 liver cancer**

Therefore, the correct Fig. 13 should be as follows:

**Fig. 13:**
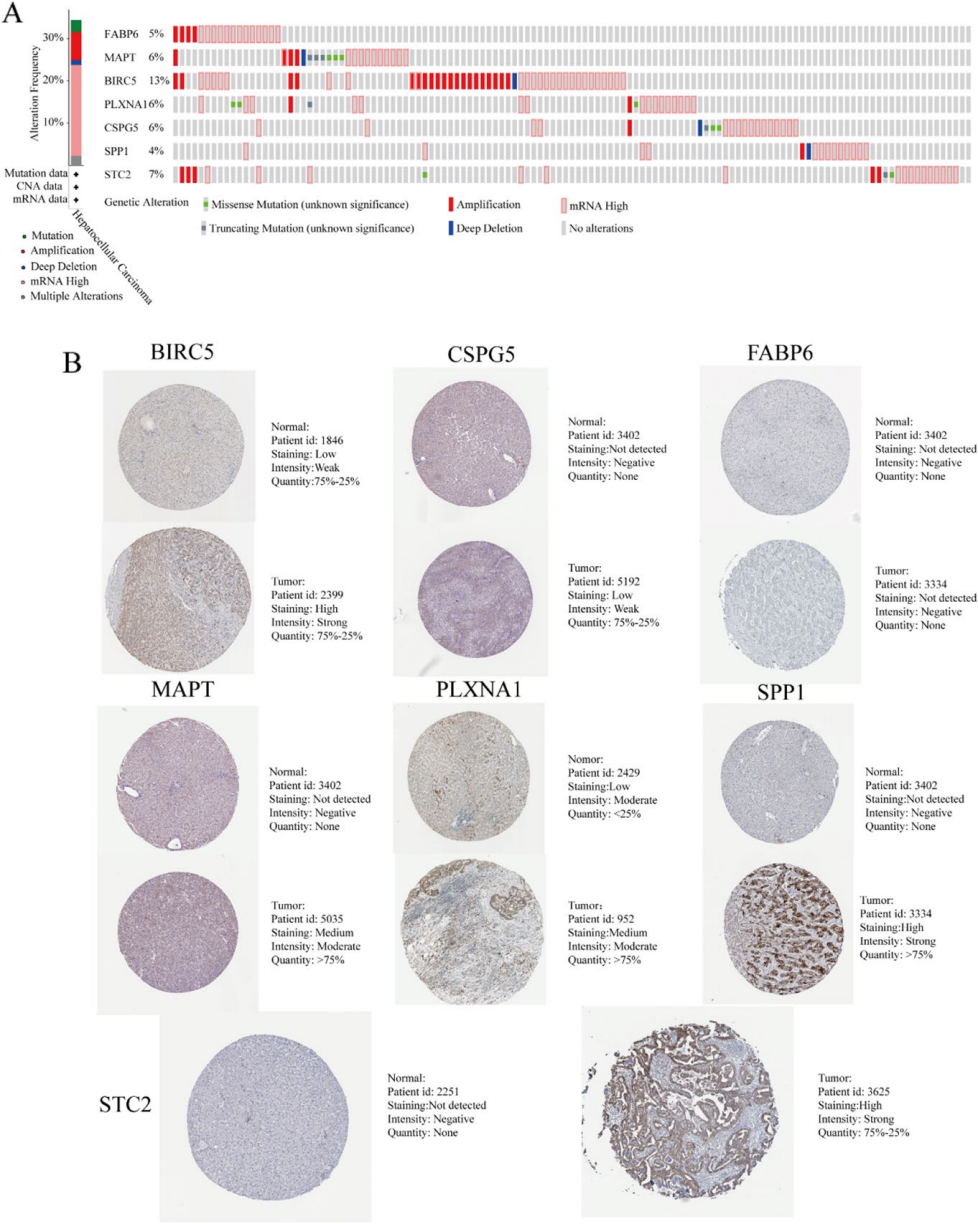
Genetic alterations landscape (**a**) and expression in the translational level (**b**) of the seven-prognostic immune-related genes in hepatocellular carcinoma
